# Improving adherence to immunosuppression after liver or kidney transplantation in individuals with impairments in personality functioning – A randomized controlled single center feasibility study

**DOI:** 10.3389/fpsyg.2023.1150548

**Published:** 2023-03-09

**Authors:** Jolana Wagner-Skacel, Nadja Fink, Judith Kahn, Nina Dalkner, Emanuel Jauk, Susanne Bengesser, Marco Mairinger, Gerhard Schüssler, Christoph Pieh, Vanessa Stadlbauer, Alexander H. Kirsch, Sabine Zitta, Alexander R. Rosenkranz, Peter Fickert, Peter Schemmer

**Affiliations:** ^1^Department of Medical Psychology, Psychosomatics, and Psychotherapy, Medical University of Graz, Graz, Austria; ^2^University Transplant Center Graz, Medical University of Graz, Graz, Austria; ^3^Division of Abdominal, Visceral, and Transplant Surgery, Department of Surgery, Medical University of Graz, Graz, Austria; ^4^Department of Psychiatry and Psychotherapeutic Medicine, Medical University of Graz, Graz, Austria; ^5^Department of Psychosomatic Medicine, University for Continuing Education Krems, Krems an der Donau, Austria; ^6^Division of Gastroenterology and Hepatology, Department of Internal Medicine, Medical University of Graz, Graz, Austria; ^7^Division of Nephrology, Department of Internal Medicine, Medical University of Graz, Graz, Austria

**Keywords:** liver transplantation, kidney transplantation, adherence, immunosuppression, multilevel intervention

## Abstract

**Introduction:**

Although adherence to immunosuppressive medication is the key factor for long-term graft survival today, 20–70% of transplant recipients are non-adherent to their immunosuppressive medication.

**Objective:**

A prospective, randomized, controlled single-center feasibility study was designed to evaluate the impact of a step guided multicomponent interprofessional intervention program for patients after kidney or liver transplantation on adherence to their immunosuppressive medication in daily clinical practice.

**Materials and methods:**

The intervention consisted of group therapy and daily training as well as individual sessions in a step guided approach. The primary endpoint of the study was adherence to immunosuppression as assessed with the “Basel Assessment of Adherence to Immunosuppressive Medications Scale” (BAASIS). The coefficient of variation (CV%) of Tacrolimus (TAC) through levels and the level of personality functioning was a secondary endpoint. We conducted six monthly follow-up visits.

**Results:**

Forty-one age- and sex-matched patients [19 females, 58.5 (*SD* = 10.56) years old, 22 kidney- and 19 liver transplantation] were randomized to the intervention- (*N* = 21) or control-group (*N* = 20). No differences between intervention- and control groups were found in the primary endpoint adherence and CV% of TAC. However, in further exploratory analyses, we observed that individuals with higher impairments in personality functioning showed higher CV% of TAC in the controls. The intervention might compensate personality-related susceptibility to poor adherence as evident in CV% of TAC.

**Discussion:**

The results of the feasibility study showed that this intervention program was highly accepted in the clinical setting. The Intervention group could compensate higher CV% of TAC after liver or kidney transplantation in individuals with lower levels of personality functioning and non-adherence.

**Clinical trial registration:**

ClinicalTrials.gov, identifier NCT04207125.

## 1. Introduction

Following solid organ transplantation, non-adherence to immunosuppressant medication is associated with poor clinical outcome including graft rejection, which leads to increased care cost (Vlaminck et al., 2004; Pinsky et al., 2009; De Geest et al., 2020). In 2019, 720 solid organ transplantations were performed in Austria, 108 of them in Graz, which included mostly kidney (KT) and liver transplantations (LT) (ÖBIG-Transplant, 2019). With 87.7 transplanted patients per million inhabitants, Austria has one of the highest transplantation rates in Europe (European Directorate for the Quality of Medicines and Health Care of the Council of Europe, 2018). For patients, transplantation is often a step into a new life after living with a chronic disease for years. However, it should not be forgotten that patients are still chronically ill (Erim et al., 2013). After KT or LT, immunosuppressive medication is required to prevent rejection. Lifelong adherence, the extent to which the patient’s behavior matches prescriber’s recommendations, to immunosuppressive medication is important to prevent graft failure (Pabst et al., 2015; Nöhre et al., 2018). Nonetheless, many transplant recipients have difficulties when it comes to medication intake. Between 20 and 70% of all transplant recipients do not follow therapy recommendations and do not take their medication as prescribed (Massey et al., 2013, 2015; Neuberger et al., 2017; Low et al., 2019). Non-adherence is linked to poor post-transplant outcomes including late acute rejection and graft loss (Dew et al., 2008; De Geest and Dobbels, 2010). Results from a meta-analysis of 147 transplantation studies show that non-adherence was the highest among kidney transplant recipients, reaching 36 cases per 100 patients per year (Dew et al., 2007). Non-adherence can be detected by objective direct measures (observation that medication was taken) or indirect (serum drug levels, biological markers, and electronic monitoring) and subjective measures such as self-reports. Adherence is a dynamic process with the need to be measured repeatedly over time. Monitoring should be incorporated into the routine clinical management of all transplant recipients (Neuberger et al., 2017; Gustavsen et al., 2019).

Risk factors for non-adherence can be categorized into five interrelated areas: socioeconomic, patient-related, disease-related, treatment-related and factors related to the healthcare setting (World Health Organization, 2003). Interventions should target more than one risk factor by combining educational and behavioral interventions over time with a multilevel approach, thereby influencing not only the patient but also the healthcare provider (Neuberger et al., 2017). Improving adherence to the immunosuppressive drug regimen is the most important intervention to improve long-term transplantation outcome (Pinsky et al., 2009; Shemesh et al., 2018).

In recent studies, the main factors influencing adherence were the knowledge about the medication, complexity of the medication, and the side effects (Zhu et al., 2017). Adherence also hinges on the relationship to the caregiver, mental illness, social support, and sociodemographic parameters. These factors are very likely influenced by the level of personality functioning and the attachment style (Mathes et al., 2017). Personality functioning describes enduring maladaptive patterns of emotion, cognition, regulation and behavior including abilities in interpersonal functioning as well as coping strategies and the regulation of affect and stress. The concept of personality structure or personality functioning–also referred to as structural integration or personality organization–describes basic self- and other-related affect-laden processing and regulatory capacities (Hörz-Sagstetter et al., 2018). Structure refers to the availability of mental functions. The concept of personality structure has its origins in psychoanalytic/psychodynamic theory and is traced back to Freud’s structural model of what he called the psychic apparatus (Freud, 1900, 2000). Personality functioning at a well-integrated level is characterized by a coherent sense of self, flexible functioning even under stress from external or internal conflicts, appropriate expression and regulation of impulses and emotions, internalized moral values, and engagement in satisfying relationships (Zimmermann et al., 2012). In the clinical environment, patients with a lower level of personality function are often experienced as “difficult to treat” (Ehrenthal et al., 2019), with the result that these patients often do not receive adequate treatment. Difficulties in the doctor-patient relationship are reflected in non-adherence and a worsening of the outcome.

Most studies that evaluated interventions targeted at adherence in adults combine educational and behavioral components and found larger effects than studies with only one component (Foster et al., 2018). The multicomponent TAKE-IT intervention, which combines electronic adherence monitoring, problem-solving skills training, and technology-based adherence support in adult kidney transplant recipients resulted in a significantly better medication adherence than in the control group (De Bleser et al., 2009). Even better effects were observed in studies, which took an individualized approach or used more frequent interventions (Bender et al., 2011). However, as several studies have shown, final recommendations on a certain adherence intervention cannot be made so far, and further research is urgently needed especially translated into daily clinical practice (Zhu et al., 2017; Duncan et al., 2018; Foster et al., 2018; Kostalova et al., 2022).

Therefore, we developed a step guided multicomponent (combining education, motivational interviewing, and psychodynamic therapy) interprofessional (consisting of psychiatrists, psychotherapists, nursing scientists, nurses) intervention to increase adherence to medical and behavioral recommendations in liver or kidney transplant recipients. The multilevel intervention program is integrated into daily routines using clinically feasible methods of screening and tracking adherence and activities that empower patients in order to improve their self-management.

In the present study, we assessed whether this approach is feasible (Tickle-Degnen, 2013) in a clinical setting and whether it improves adherence as measured by the “Basel Assessment of Adherence to Immunosuppressive Medications Scale” (BAASIS) and the coefficient of variation (CV%) of TAC-through levels. In our experience, there is a strong influence of personality functioning on emotional regulation, the doctor-patient relationship and consequently health management. Thus, we assessed the association between personality functioning and adherence to have a focus on non-adherence.

## 2. Materials and methods

**FIGURE 1:**
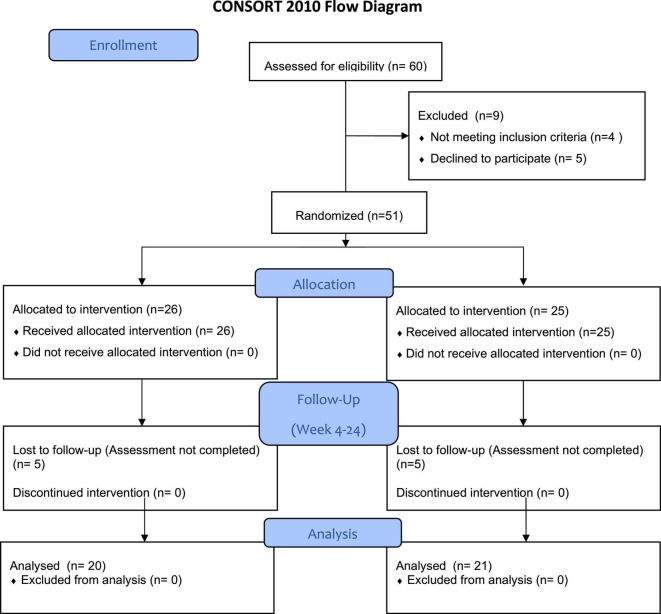
CONSORT flow diagram of the clinical trial.

**FIGURE 2:**
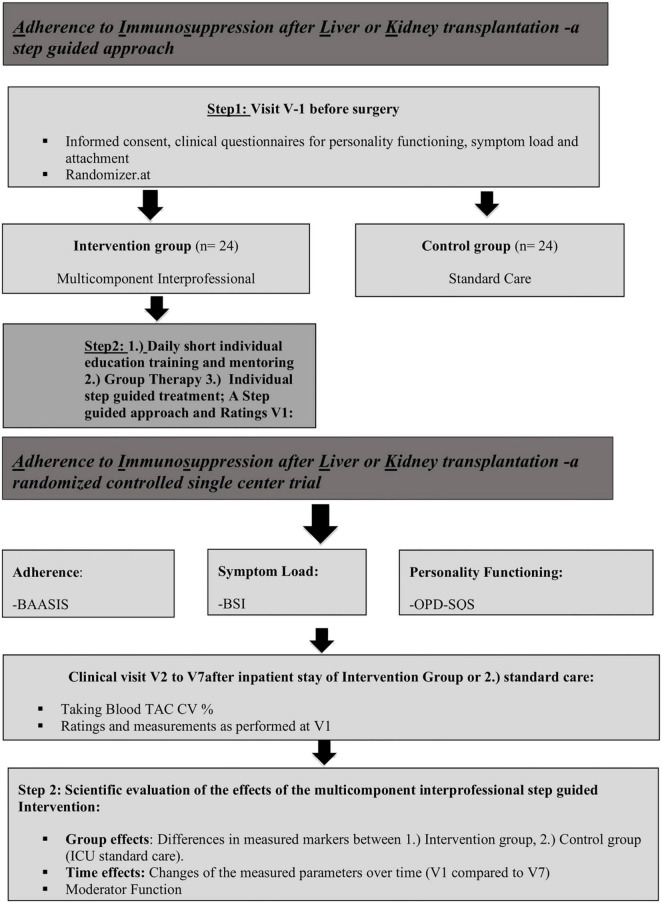
Description of the multicomponent interprofessional step guided approach.

This study was a prospective, single center, non-blinded, randomized controlled psychotherapeutic trial with two parallel groups assessing the potential superiority of a multilevel intervention program. A stratified randomization based on the type of allograft (KT or LT) was used. Depending on the stratification, the patients were randomized in a 1:1 ratio to the group receiving a multilevel intervention program during the time either after transplant or to the control group, receiving standard of care after being transplanted (shown in Figure 1) and the description of the multicomponent interprofessional step guided approach (shown in Figure 2).

A sample of 60 patients was recruited during time on the waiting list. The study was conducted at the University Transplant Center Graz, Medical University of Graz, Austria. The study was approved by the Ethics Committee of Medical University of Graz (protocol No: EK 32-062 ex 19/20).

Patients were included when they had the alarm for transplantation for LT or KT living donation, were able to understand the character and individual consequences of the trial, were fluent in German language, gave written informed consent before enrolment in the trial and received maintenance immunosuppression with TAC.

Patients < 18 or > 90 years or pregnant or lactating women were excluded. Patients waitlisted for KT or LT were approached by study personnel and were included in the study after having provided oral and written informed consent. A stratified randomization based on the organ the patient got transplanted is used. Depending on the stratification, the patients are randomized at a 1:1 ratio into the group receiving a multilevel step guided intervention program during the time either after liver or kidney transplantation or into the control group receiving standard of care after being transplanted. The online tool Randomizer (randomizer.at) was used for randomization. Clinical data were recorded, the psychological assessment and BAASIS was performed, and laboratory parameters as well as TAC through levels were recorded from the hospital database at each visit.

### 2.1. Primary outcome measure

The primary outcome was the proportion of patients categorized as non-adherent. Medication adherence was assessed at months 1–6 after transplantation, using a validated version of the Basel Assessment of Adherence to Immunosuppressive Medications Scale (BAASIS) questionnaire. The BAASIS was developed to assess adherence to immunosuppressive drugs in adult transplant recipients and followed the newly published taxonomy of medication adherence. This self-reported interview consists of three quantifiable phases: initiation, implementation and persistence. Five items assess the implementation dimension and one item assesses the persistence. An optional item assesses initiation (Dobbels et al., 2010).

### 2.2. Secondary outcome measures

Coefficient of variation (CV%) of TAC was calculated based on its through level, measured during the first 6 months after transplantation (Shuker et al., 2015). Clinical outcomes including incidence of infections, incidence of biopsy proven acute rejection, transplant function (creatinine, estimated glomerular filtration rate), death, graft losses, hospital readmissions, side effects, number of trough level controls, and achievement of TAC target concentrations during 6 months after transplantation were recorded.

### 2.3. Further patient characteristics

Personality functioning was assessed with the short version of the Operationalised Psychodynamic Diagnosis Structure Questionnaire (OPD-SQS) at inclusion of the patient and at months 1–6 after transplantation. Attachment dimensions were assessed with Experiences in Close Relationships-Revised (ECR-RS) at inclusion of the patient and at months 1–6 after transplantation.

#### 2.3.1. Intervention group

To improve adherence after transplantation, a multilevel step guided intervention program based on theoretical research including education, motivational interviewing and psychodynamic therapy in an interprofessional setting consisting of psychiatrist, psychotherapist, nursing scientists, nurses was developed and implemented at the University Transplant Center, Medical University of Graz. The first and the second part were conducted during the inpatient stay after transplantation. The third part was conducted during the transplant recipient’s outpatient follow-up appointments. A short, detailed description of the intervention program follows:

##### 2.3.1.1. Part 1 individual educational training and mentoring

After being transferred to the intermediate care unit, patients received short training units (5–10 min per day) by the nursing staff depending on the patient’s cognitive abilities. The patients were informed on currently prescribed medications and received written information about the multilevel intervention program, medication names and pictures of the medication, effects and side effects of the medication. The nursing staff at the intermediate care unit was trained in motivational interviewing and teaches back method.

##### 2.3.1.2. Part 2 group therapy

The 90 min group session with the focus on a structure based psychodynamic therapy was conducted in the first 2 weeks of the inpatient stay with the nursing scientist and psychiatrist and psychotherapist. Patients were introduced to mindfulness training, existential flourishing, stress coping strategies. Important was also the Introduction to a daily routine and day structure as a cornerstone of adherent behavior. Questions like the following are reflected in the group as a matter of what we think we are doing in our daily lives and interactions: What is to live well? What type of effort must we put in? What, when it comes to becoming ourselves, are we working with? How do others factor in? What is the role of justice in all of this? Afterward an advanced practice nurse for transplant care explained signs and symptoms of rejection and the importance of timely intake of immunosuppressive medications. Effects and side effects of the current medication were discussed with each patient and patients were instructed in the dispensation of their individual medication.

##### 2.3.1.3. Part 3 individual treatment approach

The goal of this session was to promote patient engagement in self-management of their chronic illness, to improve the patients’ ability to manage symptoms, treatments, physical and psychosocial consequences and lifestyle changes.

#### 2.3.2. Control group

Patients in the control group were treated according to standard of care and did not receive any additional intervention regarding their adherence behavior. This standard included monthly appointments with the liver or kidney transplant treatment team to assess kidney and liver function and to address any issues raised by the provider or the patient.

### 2.4. Inventories

#### 2.4.1. Basel assessment of adherence to immunosuppressive medications scale (BAASIS)

The BAASIS was used to assess adherence to immunosuppressive medications in adult transplant recipients and is available as questionnaire as well as interview guideline in several languages (Leuven Basel Research Group, 2019). Psychometric properties were tested by Marsicano Ede et al. (2013). The BAASIS consists of five items, four of which assess issues with the implementation and one the non-persistence of immunosuppressive medication use. Three items have a sub-question regarding the frequency of occurrence. Any “yes” on any of the items 1a, 1b, 2, 3, or 4 indicates that the study participant is non-adherent (Leuven Basel Research Group, 2019). Since this dichotomous scoring of the BAASIS resulted in limited variance (with partially only single participants being classified as non-adherent; see Table 2) and discards part of the assessed information, we also used a complemental, metric scoring. For this, we used the sum of items 1–4, which resulted in higher variance [see Table 2; Dobbels et al. (2010)].

#### 2.4.2. Brief symptom inventory-18 (BSI-18)

The BSI-18 was used to assess psychiatric symptoms and psychological distress in the preceding week. The inventory comprises 18 items and assesses psychological distress on the three subscales depression, anxiety, and somatization. The subscales show an internal consistency with a Cronbach’s alpha of α = 0.79 for the sub-dimensions (Derogatis, 2001).

#### 2.4.3. Operationalised psychodynamic diagnosis structure questionnaire short version (OPD-SQS)

The OPD-SQS was used as a screening instrument for supporting therapeutical decision making in treatment planning and therapy focus (Ehrenthal et al., 2015). The OPD-SQS consists of 12 Items with three subscales (self-perception, contact, relationship) explore patient characteristics, which might be of relevance to adherence, such as the level of personality functioning as self-regulatory and interpersonal competencies, would impact the effectiveness of the intervention. The subscale “self-perception” combines aspects of self with structural skills of emotion regulation. The subscale “contact” combines interactional skills with aspects of self-uncertainty. The subscale “relationship” depicts the representation of relationship experiences and connections to expectations of new relationships. The range reaches from 0 (“highest structural level”) to 48 (“lowest structural level”). The internal consistencies range from α = 0.87 to 0.89 (Ehrenthal et al., 2012).

#### 2.4.4. Experiences in close relationships-revised (ECR-RS)

The ECR-RS was used to assess differences with respect of attachment-related anxiety. It identifies four types of attachment including secure, preoccupied, detached and fearful attachment, which correspond to the secure, ambivalent, avoidant, and disorganized attachment types described by Ainsworth (1978). It contains attachment-related anxiety and avoidance features in four kinds of relationships: relationships with mother, father, romantic partners, and friends. The ECR-RS contains nine items assessing attachment in each of those four domains, therefore producing 36 items. Romantic attachment is associated with basic aspects of relationship functioning (Fraley et al., 2011). High scores indicate insecure adult attachment styles, while low scores can be viewed as having a secure adult attachment style (Brennan et al., 1998). It employs a 7-point Likert scale (*1* = “absolutely disagree” to *7* = “absolutely agree”).

#### 2.4.5. Coefficient of variation of tacrolimus (CV% of TAC)

The CV% of TAC through levels was calculated as the ratio of the standard deviation (o’) to the mean (μ) (CV percentage = o’ /μ × 100). It is a useful method for the quantification of intrapatient variability and it shows the degree of variation (Shuker et al., 2015). High intrapatient variability of tacrolimus has shown to be associated with poor outcome and higher risk for rejection (Shuker et al., 2015; Gueta et al., 2018; Rayar et al., 2018; Rahamimov et al., 2019).

## 3. Results

### 3.1. Description of the sample

The final sample with complete data sets consisted of 41 individuals, 21 of whom were randomized to the intervention group, and 20 to the control group (see also Table 1). Overall, 19 women and 22 men with a mean age of 58.49 years (*SD* = 10.56) took part in the study. The sex ratio did not differ across intervention and control groups (χ^2^_1_ = 0.30, *p* = 0.87), and no differences were found in age (*t*_37_ = −0.04, *p* = 0.97). Among the study patients, 22 underwent KT, and 19 underwent LT; this ratio did also not differ between intervention and control groups (*χ^2^_1_* = 0.21, *p* = 0.65). The patients included in the study had no adverse events. Significant correlation was found between personality functioning (OPD-SQS) and symptom load (BSI-18) a low level of structural integration was accompanied by a higher symptom load (see in Table 2).

**TABLE 1:** Sample characteristics.

	Intervention	Control	Difference test
**Demographic characteristics**			
Sample size *N*	21	20	
Sex (F/M)	10/11	9/11	*χ^2^_1_* = 0.30, *p* = 0.87
Age	58.43 (11.57)	58.56 (9.57)	*t*_37_ = −0.04, *p* = 0.97
Transplantation type (kidney/liver)	12/9	10/10	*χ^2^_1_* = 0.21, *p* = 0.65
**Psychological patient characteristics**			
Personality functioning (OPD-SQS)	11.26 (8.81)	7.00 (5.02)	*t*_28.25_* = 1.84, *p* = 0.08
Self-perception	1.00 (1.34)	2.05 (3.17)	*t*_23.95_* = 1.34, *p* = 0.19
Contact	3.63 (3.06)	2.20 (1.77)	*t*_28.49_* = 1.78, *p* = 0.09
Relationship	5.58 (4.02)	3.80 (2.95)	*t*_37_ = 1.80, *p* = 0.08
Symptom load (BSI-18)	5.16 (4.43)	7.50 (5.84)	*t*_37_ = −1.41, *p* = 0.17
Somatization	1.39 (1.09)	2.45 (2.37)	*t*_27.31_ = −1.80, *p* = 0.08
Depression	1.39 (2.85)	1.47 (1.84)	*t*_35_ = −0.11, *p* = 0.92
Anxiety	2.11 (2.19)	1.89 (1.56)	*t*_35_ = 0.35, *p* = 0.73

Values in brackets denote standard deviations. OPD-SQS, Operationalized Psychodynamic Diagnosis Structure Questionnaire Short Version (higher Scores indicate more impairments in personality functioning); BSI-18, Brief Symptom Inventory 18-item version.

*Corrected for unequal variances.

**TABLE 2:** Correlations between personality functioning (OPD-SQS) and symptom load (BSI-18).

	1	2	3	4	5	6	7
**OPD-SQS (1)**							
Self-perception (2)	***0.82***						
Contact (3)	***0.81***	***0.48***					
Relationship (4)	***0.92***	***0.65***	***0.62***				
**BSI-18 (5)**	**0.38**	**0.38**	0.25	**0.34**			
Somatization (6)	0.19	0.03	0.09	0.29	***0.64***		
Depression (7)	***0.54***	***0.70***	**0.39**	**0.37**	***0.66***	0.20	
Anxiety (8)	**0.34**	0.18	**0.39**	0.31	***0.71***	0.23	**0.38**

*N* ≥ 36. Correlation printed in bold are significant at *p* < 0.05, correlations printed in bold and italic are significant at *p* < 0.01. OPD-SQS, Operationalized Psychodynamic Diagnosis Structure Questionnaire Short Version (higher scores indicate more impairments in personality functioning); BSI-18, Brief Symptom Inventory 18-item version.

### 3.2. Statistical analyses: Main effects of the intervention

In the following, we report tests of intervention effects for our primary and secondary outcome measures. We use univariate statistical tests for the six timepoints rather than multivariate tests because outcome data were not available for each patient and timepoint, and our aim was to preserve the largest possible sample size.

#### 3.2.1. Primary outcome: Adherence assessed by the BAASIS

**FIGURE 3:**
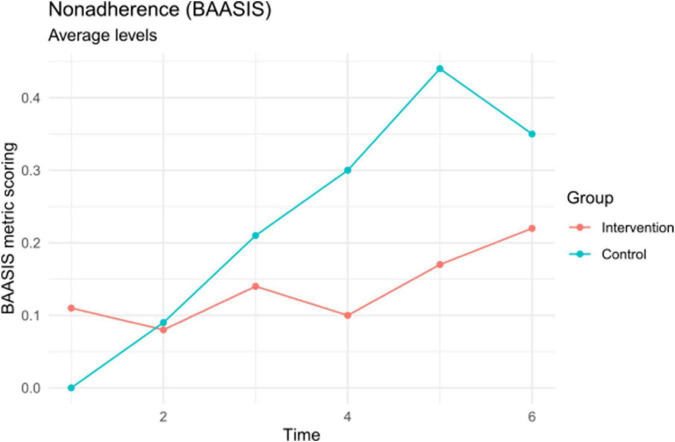
Non-adherence as assessed by the basel assessment of adherence to immunosuppressive medications scale (BAASIS) (metric scoring). Mean differences are displayed for descriptive purposes and not statistically significant (see Table 2).

To assess the effectiveness of the intervention with respect to patients’ adherence, we first evaluated differences in the BAASIS scores at each of the six timepoints. As detailed in the methods section, we used (a) the original dichotomous BAASIS scoring and (b) an alternative, metric scoring (given the limited variance in the original scoring). Table 3 and Figure 3 present the results of these tests. We did not observe significant differences between the intervention and control groups at any of the timepoints for either the original or the alternative BAASIS scoring. Note, however, that BAASIS scores were only available for 39–85% of the sample for the single timepoints (see Table 3). The drop-out rate was 14% (*n* = 3) in the intervention group and 15% (*n* = 3) in the control group, respectively. Adherence after 6 months (T6) was 78% (*n* = 14) at the intervention group, 22% (*n* = 4) were categorized as non-adherent. In the control group 76% (*n* = 13) were categorized as adherent and 24% (*n* = 4) as non-adherent. There was no statistically significance between the two groups (*X*^2^ = 0.01; *p* = 0.93). The same pattern of results was observed across other study time points. In the metric BAASIS scores we found no statistically significant differences between the intervention and control groups (see Table 3). The response rate for the BAASIS scores was low from T1 to T5 (39 to 85%, see Table 3).

**TABLE 3:** Statistical tests of intervention main effects.

	Intervention		Control					Intervention	Control		
	***n*** **_adherent_**	***n*** **_nonadh_.**	***n*** **_adherent_**	***n*** **_nonadh_.**	**χ^2^ (1)**	***p***		***x*** **_int_ (*SD*)**	***x*** **_con_ (*SD*)**	***t*** **(*df*)**	***p***
BAASIS–dichotomous							BAASIS–metric				
T1	7	2	8	1	0.40	0.53	T1	0.11 (0.33)	0.00 (0.00)	1.00 (8.00)*	0.33
T2	8	1	6	1	0.36	0.85	T2	0.08 (0.29)	0.09 (0.30)	−0.62 (21)	0.95
T3	11	3	10	3	0.11	0.92	T3	0.14 (0.36)	0.21 (0.43)	−0.48 (26)	0.64
T4	9	1	8	2	0.39	0.53	T4	0.10 (0.32)	0.30 (0.67)	−0.85 (18)	0.41
T5	11	1	6	3	2.06	0.15	T5	0.17 (0.58)	0.44 (0.73)	−0.98 (19)	0.34
T6	14	4	13	4	0.01	0.93	T6	0.22 (0.43)	0.35 (0.61)	−0.74 (33)	0.46
							Tac COV	29.63 (16.08)	30.99 (11.23)	−0.31	0.76

For the BAASIS, frequencies in the left part of the table add up to the n for the right part of the table.

*Corrected for unequal variances.

#### 3.2.2. Secondary outcome: Tacrolimus coefficient of variation

**FIGURE 4:**
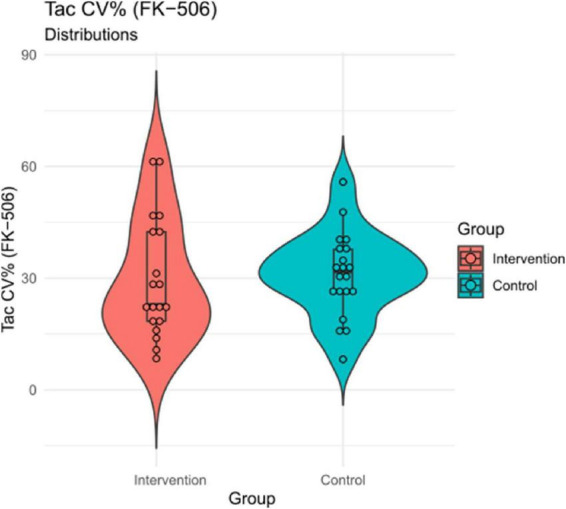
TAC coefficient of variation (CV%) by group. Mean differences are displayed for descriptive purposes and not statistically significant (see Table 2).

To assess the effectiveness of the intervention with respect to variation in TAC levels across the study period, we used the coefficient of variation (across all timepoints, assuming a stable TAC target level in the early post-transplant phase) as secondary outcome measure in a between-groups comparison. The analysis did not yield evidence for a significant intervention effect at a between-groups level [*x*_*int*_ = 29.63 (16.08), *x*_*con*_ = 30.99 (11.23); *t*_37_ = −0.31, *p* = 0.76; see Table 3 and Figure 4].

### 3.3. Exploratory analyses: Impact of patient characteristics on intervention effectiveness

Our statistical analyses did not yield evidence for a main effect of the intervention on the primary outcome measures; however, it might be the case that individuals benefit differentially from the intervention. As outlined above, particularly those individuals with lower levels of personality functioning might benefit more from the intervention. To investigate the potential impact of personality functioning on the intervention effectiveness, we first inspected correlations of personality functioning (OPD-SQ) and outcome measures separately for treatment and control groups. This might give hints on whether the associations between personality functioning and the outcome measures differs between the groups, or, in other words, whether the intervention effectiveness depends upon patient characteristics. We then tested the significance of differences in those coefficients which displayed notable differences in the first place using a formal moderation analysis (multiple regressions).

**FIGURE 5:**
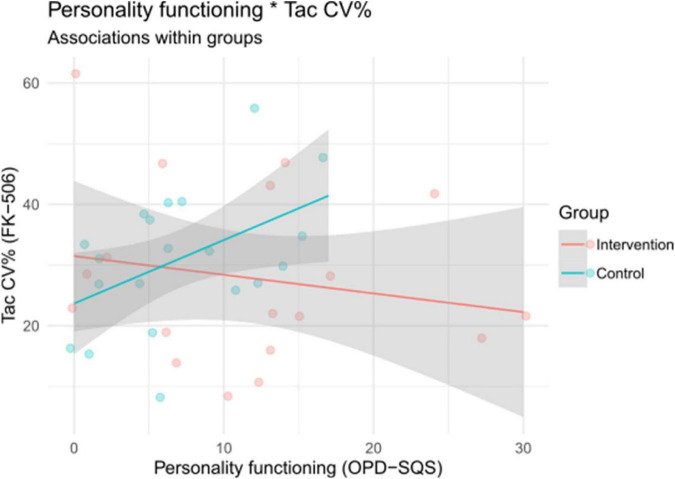
Associations between personality functioning and TAC coefficient of variation (CV%) within groups. OPD-SQS, Operationalized Psychodynamic Diagnosis Structure Questionnaire Short Version.

We observed a notable difference in correlations between groups in the relation of OPD-SQS and TAC COV between groups (*r*_*int*_ = –0.19, *p* = 0.46; *r*_*con*_ = 0.47, *p* = 0.04; Δ*r* = 0.66). As Figure 5 shows, there was a strong and significant positive relationship between personality functioning and TAC COV in the control group, which means that those with higher impairment in personality functioning displayed higher variation in TAC. Such an associations was not evident in the intervention group, where the correlation was weak and non-significant. The TAC COV was thus not dependent upon personality functioning in patients in the intervention group. A formal test of moderation showed that the difference in magnitude of these correlations is statistically significant (interaction test; see Table 4). We also observed a difference of Δ*r* = 0.37 between OPD-SQS and average BAASIS scores (*r*_*int*_ = 0.47, *p* = 0.05; *r*_*con*_ = 0.10, *p* = 0.69), but this interaction was not statistically significant (see Table 4).

**TABLE 4:** Interaction tests.

	Criterion	
	**BAASIS metric avg.**	**Tac COV**
Model 1: Main Eff.		
Intervention	β = 0.17, *p* = 0.32	β = 0.13, *p* = 0.47
OPD-SQS	**β = 0.35, *p* = 0.05**	β = 0.03, *p* = 0.88
Model 2: + Interact.		
Intervention	β = 0.18, *p* = 0.31	β = 0.12, *p* = 0.47
OPD-SQS	**β = 0.34, *p* = 0.05**	β = 0.02, *p* = 0.90
Intervention*OPD-SQS	β = –0.11, *p* = 0.50	**β = 0.33, *p* = 0.05**

Variables linked with * represent interaction term. Coefficients printed in bold are significant at *p* < 0.05. OPD-SQS, Operationalized Psychodynamic Diagnosis Structure Questionnaire Short Version.

To further explore the nature of the correlation differences in the association of the OPD-SQS and the TAC COV between the control and intervention groups, we repeated the aforementioned correlation comparisons (control vs. interventions group) for the OPD-SQS subscales *self-perception*, *contact*, and *relationship*. This might give hints on which aspects of personality functioning impact the intervention effectiveness. We observed equal differences in correlation of Δ*r* = 0.62 for the *contact* (*r*_*int*_ = −0.05, *p* = 0.86; *r*_*con*_ = 0.57, *p* = 0.01) and *relationship* subscales (*r*_*int*_ = −0.33, *p* = 0.19; *r*_*con*_ = 0.29, *p* = 0.21), the difference in correlation for the *self-perception*–subscale was somewhat smaller (*r*_*int*_ = −0.06, *p* = 0.82; *r*_*con*_ = 0.36, *p* = 0.12; Δ*r* = 0.52). This points to the impact of interpersonal aspects of personality functioning for intervention effectiveness.

## 4. Discussion/Conclusion

This feasibility study presents a randomized controlled single-center trial using a multilevel intervention program for improving medication adherence in patients after LT or KT implemented in a clinical setting. We did not find differences in adherence measured with BAASIS between intervention- and control group. We observed a notable difference in correlations between groups in the relation of level of personality functioning and TAC COV. Without intervention, individuals with impairments in personality functioning had higher TAC COV values. The intervention is able to compensate these individual differences in personal vulnerability. A formal test of moderation showed that this interaction was statistically significant. We found the measurements and interventions well-accepted with high completion rates in a cohort of 41 patients LT or KT, respectively. Our most important finding is a significant correlation of personality functioning PF and CV% of TAC with improvement in individuals that would have difficulties in adherence.

The focus on patients with non-adherence was recently published to be a goal in the management of adherence in a multidisciplinary team with the use of novel therapeutic approaches focus on multimodal therapy for non-adherent population incorporated in a realistic clinical setting (Myaskovsky et al., 2018; Geramita et al., 2020; Kuypers, 2020). Individuals with lower levels of personality functioning might benefit more from the intervention program because of the frequent contact and the training of behavioral changes with the goal to improve health literacy and the attitude toward oneself. Personality functioning levels are thought to vary on a continuum ranging from unimpaired/well-integrated to severely impaired/disintegrated (Clarkin and Huprich, 2011). Personality functioning at a well-integrated level is characterized by a coherent sense of self, flexible functioning even under stress from external or internal conflict, appropriate expression and regulation of impulses and emotions, internalized moral values, and engagement in satisfying relationships (Zimmermann et al., 2012). Individuals at lower levels of personality functioning typically exhibit problems with self-regulation or self-other differentiation. This ability comes with a number of associated challenges and has implications for unhealthy behavior and interpersonal relationships, including the doctor-patient relationship (Stern et al., 2010; Wagner-Skacel et al., 2021). We see this link between low levels of personality functioning and symptom load including depressive, anxiety and somatization symptoms in transplant recipients. These finding underline the increasing importance of assessing personality functioning for diagnosis and treatment planning.

There might be several reasons why we did not observe a difference in adherence between intervention- and control groups. On the one hand the reason could be the small sample size in our study, on the other hand the passing of the measurements as BAASIS, which might be more important to measure the progress than the outcome of an intervention study with focus on non-adherence. A systematic review and the COMMIT group recommended this validated scale as the most appropriate self-report instrument for measuring non-adherence in transplant recipients because of its simplicity and ease of scoring (Dobbels et al., 2010; Neuberger et al., 2017). Another reason might be a selection bias: patients who agreed to participate may have more openness and interest regarding education and therefore higher adherence.

Multilevel intervention programs show a long-lasting effect on improving medication adherence after transplantation (Brennan et al., 1998; Low et al., 2015; Mathes et al., 2017; Schäfer, 2017). Therefore, it is necessary to offer individual educational training, mentoring and group therapy during the inpatient stay and an individual treatment approach during the outpatient follow-up appointments. So far, there are no study findings about intervention programs which start in the immediate post-transplant period. A strength of the present intervention program is its patient-centered approach, which allows influencing factors for non-adherence to be identified and addressed early as the implementation in a real -word setting. Much of the extant literature on adherence barriers has focused on modifiable factors (e.g., knowledge, social support), however, less is known about how barriers may be associated with relatively stable constructs such as personality and attachment. The evaluation of the implementation shows associations between personality functioning and adherence. This may lead to more personalized interventions oriented on the individual needs of the patients. Personality functioning, also referred to as structural integration, describes basic emotion-related perception and regulation capacities directed toward the self and others. Patients with impairments of structural integration are detracted in their psychosocial functioning and experience difficulties in self-regulation and interpersonal relations. Social support and functioning in transplant patients are important variables guaranteeing psychological and social wellbeing (Garcia et al., 2018). The importance of social functioning has been recognized in coping with stress and health treatment adherence (Ordin and Karayurt, 2016) providing better physical and mental health effects (Langenbach et al., 2008). Social and personality functioning describes patterns of emotion, cognition, regulation, and behavior in social interactions. Patients impaired in their social and personality functioning are more skeptic toward the treatment team and have a lack of interpersonal relations. In the clinical setting these patients are often experienced as “difficult to treat” (Ehrenthal et al., 2019). Due to the recent important change of personality disorder classifications, in a dimensional or a composite categorical dimensional approach for personality, the personality functioning and social functioning construct includes a broad range of personality facets (Zimmermann et al., 2012). In particular, the focus on domains beyond symptoms, such as global personality functioning has been accepted as highly important for indication and treatment planning (Doering et al., 2014). Perceived weak social support is an important risk factor for poor commitment to adhere to a treatment regimen (Blumenthal et al., 2006) especially among transplant providers, in determining patients’ suitability for transplantation (Ladin et al., 2018). Improving adherence is fundamentally linked to a stable relationship between physician and patient characterized by trust. This is better managed by the patient through a secure attachment style and a well-integrated personality functioning (Jennissen et al., 2020).

A structured assessment of waitlisted patients’ personality traits may be a valuable addition to routine pre-transplant data gathered. This may allow to more accurately identify patients who are at increased risk for non-adherence after transplantation and potentially provide these patients with interventions that are designed to mitigate this risk (Chan et al., 2013).

The major limitation was the open study design where participants, psychiatrist, advanced practice nurse and nurses who are performing the interventions are aware of the participant’s treatment allocation. Furthermore, the participants received information about the treatment and the intended goal, which may have led to information bias. We note that, due to the small sample size, the main confirmatory hypotheses tests might be underpowered (particularly regarding the dichotomous BAASIS scoring as primary outcome; see Table 3). Also, the exploratory analyses presented here await replication in larger samples, since the within-group sample sizes were small for correlational analyses. Still, the patients’ personality functioning as a variable with impact on adherence interventions may provide a potentially important starting point for future works. A further limitation of this study may be the assessment method of the primary outcome, which is based on a self-report of medication adherence using the BAASIS questionnaire and can lead to a self-reporting bias. Therefore, it was decided to follow recommendations to combine direct and indirect measurement methods to obtain more reliable results (Neuberger et al., 2017).

In conclusion, this study aimed to generate evidence for a clinically feasible multicomponent interprofessional step guided intervention program that fits into daily post-transplant routines with cost, time and personnel effectiveness. The novel therapeutic strategy is also tailored to the individual patient needs. The intervention program was highly accepted in a real-life setting and could compensate higher TAC COV after liver or kidney transplantation in non-adherent individuals with lower levels of personality functioning. Therefore, investigating the bio-psycho-social underpinning of non-adherence and its treatment is crucial to improve live-saving adherence. We also explored whether patient characteristics, which might be of relevance to adherence, such as the level of personality functioning as self-regulatory and interpersonal competencies, would impact the effectiveness of the intervention. The study findings may also have relevance to other patient groups with chronic conditions in whom medication non-adherence contributes to negative outcomes.

## Data availability statement

The raw data supporting the conclusions of this article will be made available by the authors, without undue reservation.

## Ethics statement

The studies involving human participants were reviewed and approved by Local Ethics Committee of the Medical University of Graz, Austria (EK 32-062 ex 19/20). The patients/participants provided their written informed consent to participate in this study.

## Author contributions

JW-S: conceptualization, data curation, and writing—original draft. NF and JK: conceptualization and data curation. ND: conceptualization and methodology. EJ: methodology and writing—original draft. SB and MM: funding acquisition. GS and CP: supervision. VS and AK: data curation and supervision. SZ: conceptualization. AR and PF: supervision. PS: conceptualization and editing of manuscript. All authors contributed to the article and approved the submitted version.
